# Which Psychopathological Syndromes Could Be Associated with the Risk of Suicide among Substance Users?

**DOI:** 10.3390/ijerph15102279

**Published:** 2018-10-17

**Authors:** Laura Masferrer, Elisenda Escalé-Muntañà, Rafel Malagón, Jordi Cid, Beatriz Caparrós

**Affiliations:** 1 Public Addiction Centre, Cas Teresa Ferrer, Mental Health and Addiction Research Group, IDIBGI Institut d’Assistència Sanitària (IAS), 17002 Girona, Catalonia, Spain; jordi.cid@ias.cat; 2 Department of Psychology, University of Girona, 17004 Girona, Catalonia, Spain; Beatriz.caparros@udg.edu; 3 Gironine Helping Gambling Association (AGAL), 17002 Girona, Catalonia, Spain; eescalem@gmail.com; 4 Psicosocial Rehabilitation Unit, Institut d’Assistència Sanitària (IAS), 17190 Girona, Catalonia, Spain; rafel.malago@ias.cat

**Keywords:** complicated grief, clinical syndromes, substance use disorder, risk of suicide

## Abstract

*Background*: Research has shown that suicide is a highly present phenomenon among the drug dependent population. Moreover, individuals with substance use disorder (SUD) present high psychopathological comorbidity. This study aimed to describe which clinical syndromes are linked to the presence of risk of suicide. *Methods*: The study was based on a consecutive non-probabilistic convenience sample of 196 patients who attended the Public Addiction Center in Girona (Spain). Sociodemographic data, as well as suicide risk and drug related characteristics, were recorded. The risk of suicide was assessed with the Spanish version of “risk of suicide”. Complicated grief was assessed with the Spanish version of the Inventory of Complicated Grief. Clinical syndromes were measured with the Spanish version of MCMI-III. *Results*: The syndromes most frequently associated with the presence of risk of suicide were complicated grief, major depression and thought disorder. *Conclusions*: Different psychopathological syndromes were identified in relation to risk of suicide among patients with SUD. The present results highlight the importance of accurately diagnosing those individuals.

## 1. Introduction

According to the World Health Organization (WHO) [1,2], almost 800,000 deaths were attributed to suicide worldwide in 2016, which implies one death every 40 s. This data depicts an annual percentage rate of suicide, adjusted by age, of 10.5 per 100,000 inhabitants [3] (1.8; majority male suicides). Thus, suicide represents the eighteenth leading cause of death worldwide for all ages and the second leading cause of death for those aged 15–29. It is relevant to remark on the fact that suicide as a continuum includes suicidal ideation, planning, attempts and even suicide completion [4]. The WHO indicate a prior suicide attempt as the most important risk factor for suicide in the general population [2] and also notice the association between suicide and mental disorders (in particular, depression and alcohol use disorders). Taking this into account, some researchers have noted that around 40% of patients in treatment for substance use disorders (SUDs) report a history of previous suicide attempts [5,6,7,8]. Different previous studies underline, in the same line as the WHO, SUD as a risk factor of suicide [9,10,11,12,13,14]. Considering the suicide disability-adjusted life years that are attributable to mental and substance use disorders, alcohol dependence was responsible for the second largest proportion (13.2%), only behind major depressive disorder [15]. In fact, nearly 90% of all deaths by suicide are associated with a mental health diagnosis or SUD [16]. Moreover, in relation to the link between SUD and suicide death, SUD is especially associated with an increased risk of suicide ideation and suicide attempts [17]. An Iranian meta-analysis [18] found that alcohol use disorder and the harmful use of alcohol significantly heightened the risk of suicidal ideation, suicide attempts and completed suicides. In addition, alcohol use disorder is also an important predictor of suicide and a large cause of premature death [18,19]. Up to 40% of individuals with alcohol dependence attempt suicide at some time in their life and 7% end their lives dying by suicide [20]. According to Pan and collaborators [21], those individuals with a dependence on heroin are more likely to die by suicide compared to the general population. A recent study points out that all substances are associated with suicidal ideation, and opioids and polydrugs are related with prior suicide attempts. [14]. In relation to cocaine, the prevalence of cocaine consumption days before suicide varies from 9.4% to 20% [22]. Moreover, almost half (43.5%) of cocaine dependent patients in treatment (*n* = 406) reported suicide attempts [6].

Bearing in mind that different studies find high rates of comorbid substance use and severe mental illness, it is important to emphasize the prevalence of dual diagnosis [23,24]. According to the Substance Abuse and Mental Health Services Administration (SAMHSA) [25], approximately 7.9 million adults in the USA have co-occurring disorders. In this sense, there is a growing body of evidence that recognizes the link between mental disorders as well as substance use disorders and suicide. Some studies report that clinical disorders increase the risk of suicide attempts [15,26]. Taking this prevalence into account, literature has shown increased all-cause (natural causes, accidents, suicide and homicide) mortality among the psychiatric population. Specifically, a study by Steingrimsson [23] marks SUD as having the highest mortality of all the diagnostic groups. Several studies point out that the main factors associated with suicide attempts were: borderline personality disorder, lifetime abuse (whether emotional, physical or sexual), co-occurrent psychotic disorders, polydrug abuse, anxiety disorders and depressive symptoms [14,27,28].

Going deeper into the topic of gender in SUD patients, different studies indicate the existence of gender-specific risk factors shared by both recurrent and serious suicide attempters [27,29]. However, it seems that women in treatment present with more suicidal ideation and attempts [14]. It is relevant to note that men can be under-diagnosed with suicidal ideation and attempts because they are more reluctant to seek help [29,30]. This could explain the higher suicide mortality among men [3]. Nevertheless, previous studies reported no statistical differences between gender and risk of suicide [31] and between gender and complicated grief [32] among a SUD sample. 

In relation to comorbidity, a relationship between suicide and complications in bereavement has been described [33]. Thus, people who reported complicated grief (CG) symptoms were associated with a 9.7 times greater likelihood of suicide ideation [34]. In a similar vein, another study [33] stated that CG was linked with a 6.6 times greater likelihood of risk of suicide.

An American study [35] stated that, from their multivariate analysis, the incremental predictive effects of individual disorders are much less powerful and that a general pattern of interactive effects exists for comorbidities involving a large number of disorders. In the same way, Hoertel and collaborators [36] specify that risk of suicide is mediated by a predisposition to general psychopathology factors instead of only associated with a single disorder. This study gives support to the dimensional conceptualization of psychopathology and asks for the identification of psychological and biological processes underlying these great psychopathological dimensions. Recent latent variable modeling studies have indicated that common forms of psychopathology are best thought of as continuous, rather than categorical, in nature. Continuous models classify people by locating them along graded dimensions, whereas categorical models classify people into distinct groups [37]. As far as understanding disorders in a dimensional way, we used the Millon Multiaxial Clinical Inventory because Millon [23] proposed an explanation of the structure of psychopathology in a dimensional alternative model based on the background of ecological adaptation. Clinical syndromes are best understood as disorders rooted in the context of personality styles [38]. It allows the framing of our study and the conceptualization of psychopathological entities in a dimensional manner rather than as categorical entities.

Following on from the previous literature, the main purpose of the current study is to estimate which psychopathological syndromes are more frequently associated with risk of suicide among the SUD population.

## 2. Materials and Methods

The present research is part of a wider project. The main purpose of this study was to describe the symptoms of complicated grief among a sample of 196 bereaved SUD patients. For more information, see Masferrer and collaborators [39].

### 2.1. Participants

The current study was based on a consecutive non-probabilistic convenience sample of individuals (*n* = 196) attending the Public Addition Treatment Centre in Girona (Catalonia, North Eastern part of Spain).

The three inclusion criteria were (a) they had a diagnosis of SUD (specifically, alcohol, cocaine or heroin dependence) according to the 4th revised edition of the Diagnostic and Statistical Manual of Mental Disorders (DSM-IV-TR) [40] criteria. The authors used the DSM-IV-TR criteria instead of the DSM 5 because data collection took place from September 2012 to June 2013; (b) loss of a significant person (family, best friend or partner) at some time in their life, but at least a year previously to the interview; and (c) abstinence during the last month to avoid any toxic drug effects.

The majority of participants were male (78.1%); 30.9% reported having primary studies. In relation to the main drug diagnosis, more than a half of the patients (68.9%) reported alcohol dependence, 18.4% heroin dependence and 12.8% cocaine dependence.

### 2.2. Measures

Risk of suicide was assessed with the Spanish version [41] of the Risk of Suicide [42]. This instrument could discriminate between individuals and patients with no suicide attempts and those having a history of them. The Risk of Suicide scale consists of 15 items with dichotomous responses (yes/no). The cut-off proposed by the authors of the Spanish version [42] was 6. Internal consistency of the test is 0.90. The test–retest reliability is 0.89 (after 72 h). This instrument includes questions about previous attempts, ideation intensity of current feelings of depression and hopelessness, and other aspects of the attempts.

Clinical syndromes were measured by the Millon Multiaxial Clinical Inventory [38], using the Spanish version by Cardenal and Sánchez-López [43]. We used the most conservative criteria with prevalence scores equal to or greater than 85 to define the presence of the clinical syndromes. The MCMI-III consists of 175 items with dichotomous answers (true/false), a self-report questionnaire that measures 11 clinical personality patterns, 3 traits of severe personality pathology, 7 syndromes of moderate severity, 3 severe syndromes, along with a validity scale and 3 modifying indices. We used the direct scores of the scales as a continuous variable. The internal consistency is 0.66 to 0.80 and the test–retest reliability for dimensional ratings is 0.85 to 0.93. We used the direct scores of the scales as a continuous variable. 

Complicated grief was assessed using the Spanish version [44] of the “Inventory of Complicated Grief” (ICG) [45]. It consists of 19 items. Responses are provided on a 5-point Likert scale, indicating an increase in severity (0—never, 1—seldom, 2—sometimes, 3—often and 4—always) (range: 0–76). The cut-off point was based on the English version of the ICG [45]. We categorized a respondent as having CG if the total score was higher than 25. The internal consistency of the Spanish version was high (Cronbach’s alpha = 0.88; test–retest reliability = 0.81).

### 2.3. Procedure

Those patients who presented with the three inclusion criteria were asked by their therapist to join in the study. The research procedure consisted of a single visit with a psychologist who administered the questionnaires included in the study protocol. All patients were previously informed about the study procedure as well as the terms of confidentiality. Informed consent was obtained from all participants and the protocol was approved by the Institutional Ethics and Research Review Board of the Institut Assistència Sanitària (IAS) (No. S041-779).

### 2.4. Statistical Analyses

The risk of suicide was assessed, as well as the relative frequency and the 95% level of confidence of participants above the RS cut-off point. Because of the small group of clinical syndromes, we used a non-parametric statistical test. Specifically, we used the Kendall’s tau correlation. A hierarchical linear regression analysis was performed in order to determine which clinical syndromes were associated with the risk of suicide. We introduced the predictors in 3 steps (1. CG, 2. gender and 3. clinical syndromes). The results were expressed as absolute numbers and percentages, as well as the mean and standard deviations. A statistical significance of 0.05 was used to compare hypotheses. Data processing and analyses were performed using the SPSS statistical program version 21.0 for Windows (IBM Corp., Armonk, NY, USA).

## 3. Results

### 3.1. Psychopathological Syndromes

Table 1 provides information about the presence of disorders and the corresponding average of the direct scores (M). We adopted the most conservative criteria of Millon’s scoring [38] (scoring equal or greater than 85 prevalence score) for estimating the association of clinical syndromes as possible disorders. The most occurrent clinical syndromes among the SUD sample were anxiety (33.2%), thought disorder (13.3%) and depression (9.7%). On the other hand, there was no presence of somatoform disorders. Furthermore, the presence of CG symptomatology among the clinical sample was 34.2%.

Categorical scores (presence–absence) were used to indicate the presence of disorder, but the direct scores of the scales were employed to show the mean and standard deviations as well as by the analysis of correlations and regression.

Taking into account that the number of clinical syndromes was small, we assessed using a non-parametric statistical test (see Table 2). Our first goal was to estimate the association of psychopathological entities in relation to risk of suicide. As shown in Table 2, all the different psychopathological constructs present a statistically significant relation with risk of suicide. Moreover, the correlation with risk of suicide is in most cases with significant magnitude. This data reveals that the constructs with the highest correlation with risk of suicide were depression and thought disorder.

### 3.2. Psychopathological Constructs Associated with Risk of Suicide

The main purpose of the current study was to determine which psychopathological syndromes were associated with the risk of suicide. Table 3 provides the results of a hierarchical linear regression in which scores of risk of suicide were the dependent variable. Gender, symptoms of CG and clinical syndromes were used as independent variables. Results reported that depression, thought disorder and CG were associated with risk of suicide. In fact, depression was the clinical syndrome with the strongest contribution to explain the risk of suicide. However, somatoform disorder showed an inverse relationship with risk of suicide. Respondents who reported more symptoms of somatoform disorder also reported lower levels of risk of suicide.

## 4. Discussion

The first aim of the current research was to estimate the association of psychopathological entities in a bereaved SUD sample. The study revealed that the most frequent psychopathological syndrome was CG (34.2%) followed by anxiety (33.2%) and thought disorder (13%). More than one third of patients experienced high levels of complications in bereavement and anxiety. Previous studies have shown a strong association between anxiety and SUD [46,47,48]. In fact, as Kessler and collaborators [48] noted, anxiety and SUD are among the most frequent psychiatric problems in the United States, with lifetime rates of 28.8% and 14.6%, respectively. Moreover, the presence of both psychopathological constructs is a risk factor for the presence of the other disorder, as stated in both epidemiological and clinical samples [48,49].

It is important to analyze the relationship between anxiety and SUD for their high comorbidity, development and maintenance characteristics, and their impact on the functioning and treatment of patients who suffer from these conditions [49].

Anxiety disorders and SUD share common risk factors, such as high stress reactivity [50,51]. In relation to stress management, we can say that the deficit in emotional regulation is a risk factor for the co-occurrence of psychopathological disorders. From a transdiagnostic perspective, many emotional symptoms and psychopathological disorders are based on transversal vulnerability processes [52]. As Zvolensky and Bernstein (2005) point out in relation to the consumption of nicotine, people with high anxiety sensitivity can respond to stress by consuming substances to deal with anxiety states and the signs and symptoms that accompany it in the absence of more adaptive coping strategies [53].

The second objective was to determine the degree of the association between psychopathological entities and risk of suicide through correlation. Our findings indicated that all the clinical syndromes, as well as CG, present a statistically significant relation with risk of suicide. Specifically, our data reveals that the constructs with the highest level of correlation with risk of suicide were depression and thought disorder. These findings suggest that some clinical syndromes were more closely related to the risk of suicide in comparison to complicated grief. As mentioned in the background literature, a high level of comorbidity was reported, which underlines the broad psychopathological dimensions. These finding suggest that risk of suicide is not exclusively associated with any single disorder, but rather mediated by an overall latent influence with psychopathology [36].

The third main purpose of the current study was to determine which psychopathological syndromes were associated with risk of suicide using the hierarchical linear regression method. The results revealed that depression, complicated grief and thought disorder were associated with risk of suicide. These results corroborate the findings of a large number of previous studies which reported high correlation between depression and risk of suicide [14,54,55,56]. In the same view, the association between CG and risk of suicide broadly supports the work of others [33].

In relation to the scale of alterations of thought, our results support those obtained in the study by Robert, Craig and Bibens [57] in which they found higher average scores on this scale among patients with suicide attempts.

An unexpected finding is the negative association between the somatoform scale and risk of suicide. Previous studies found a positive association between somatoform syndrome and risk of suicide [58] or between this syndrome and suicide attempts in a sample of patients with abusive problems [57]. Other previous research also pointed in this direction, reporting a close association between the presence of somatization problems and the risk of suicide in other populations [58]. In relation to this point, we could speculate that the items that evaluate the somatoform scale of MCMI-III [38] focus particularly on specific physical problems, such as fatigue, tiredness or balance problems which can be highly occurrent among SUD patients due to the problems and consequences of substance abuse among the participants, and this fact excludes it as a scale that discriminates between those patients who have or do not have risk of suicide.

Comorbidity in psychopathology is more the norm than the exception. Therefore, it is important to analyze the psychopathological characteristics associated with different conditions in a certain disorder. Research is needed not only into comorbid disorders, but also the study of disorders from a dimensional approach that allows us to identify the underlying nuclear mechanisms and processes to different disorders and conditions (e.g., patients with or without risk of suicide).

These findings shed light on a vulnerable population who report psychopathology syndromes. Therefore, results suggested that co-occurring substance dependence and psychiatric comorbidity warrant attention among the SUD population [59].

The present study did have some limitations. The cross-sectional nature of the research does not permit the establishment of a causal relationship among the psychopathological entities, although powerful evidence does appear. We relied exclusively on self-reported measures. Moreover, the clinical syndromes were assessed with the psychometric test focus on personality disorders. Another important limitation was the sample size. However, the exploration of a small sample such as this can lead to a better understanding of their mental health. Further research may include larger samples as well as a control group.

These findings have significant implications on a therapeutic level. As Hoertel and collaborators [36] noted, interventions on individual disorders are likely to reduce the risk of suicide, but interventions directed at wider, more global psychopathological processes could have greater effect. Our findings support the current dimensional conceptualizations of psychopathology [60,61,62] and underline the necessity to identify the psychological and biological processes underlying the extensive psychopathological dimensions.

## 5. Conclusions

Our results point to the need to further study the phenomenology of psychopathology among SUD populations. Depression, complicated grief and thought disorders can be considered relevant risk factors for risk of suicide. In conclusion, these findings underline the importance of designing personalized treatments. Therefore, finding clear correlations between comorbid psychopathology for those in substance use treatment would have relevant implications for predicting treatment outcomes [63].

This greater knowledge of the underlying dimensions should allow for more specialized and integrated treatment for people with disorders due to the use of substances and other comorbidities.

## Figures and Tables

**Table 1:** Occurrence of psychopathological syndromes. PTSD = post-traumatic stress disorder. M = the average of the direct scores. SD = standard deviation.

Clinical Syndromes	Presence of Disorder (*n*, (%))	M (SD)
Anxiety	65 (33.2)	7.93 (5.2)
Somatoform	0	0
Bipolar	12 (6.1)	8.4 (3.9)
Dysthymia	9 (4.6)	6.97 (5.6)
PTSD	8 (4.1)	7.96 (6.1)
Thought	26 (13.3)	8.28 (5.8)
Depression	19 (9.7)	7.15 (6.3)
Delusional	9 (4.6)	3.61 (3.5)
Complicated grief	67 (34.2)	21.73 (16.7)

**Table 2:** Relationship between clinical syndromes and risk of suicide through Kendall’s tau correlation.

Clinical Syndromes	Kendall’s Tau Correlation	*p*
Anxiety	0.427	<0.001
Somatoform	0.417	<0.001
Bipolar	0.243	<0.001
Dysthymia	0.489	<0.001
PTSD	0.470	<0.001
Thought	0.491	<0.001
Depression	0.530	<0.001
Delusional	0.193	<0.001
Complicated grief	0.332	<0.001

**Table 3:** Hierarchical linear regression analysis of clinical syndromes associated with risk of suicide.

Clinical Syndromes	Unstandardized Coefficients		Standardized Coefficients	t	Sig.	95% Confidence Interval for B	
	B	Standard Error	Beta			Lower Bound	Upper Bound
ICG	0.092	0.0013	0.459	7.189	0.000	0.067	0.118
ICG	0.093	0.013	0.461	7.209	0.000	0.067	0.118
Gender	−0.442	0.519	−0.054	−0.053	0.395	−1.465	0.581
ICG	0.025	0.013	0.126	2.005	0.046	0.000	0.051
Gender	0.180	0.414	0.022	0.436	0.663	−0.636	0.996
Anxiety	0.030	0.066	0.046	0.450	0.653	−0.1000	0.160
Somatoform	−0.274	0.087	−0.367	−3.139	0.002	−0.446	−0.102
Bipolar	0.029	0.059	0.033	0.488	0.626	−0.087	0.144
Dysthimia	0.036	0.063	0.059	0.567	0.572	−0.089	0.160
PTSD	0.040	0.057	0.073	0.711	0.478	−0.072	0.150
Thought	0.155	0.067	0.265	2.320	0.021	0.023	0.287
Depression	0.338	0.078	0.633	4.335	0.000	0.184	0.492
Delusional	−0.077	0.063	−0.079	−1.215	0.226	−0.201	0.048
